# Beliefs associated with cancer screening behaviors among African Americans and Sub-Saharan African immigrant adults: a cross-sectional study

**DOI:** 10.1186/s12889-022-14591-x

**Published:** 2022-11-29

**Authors:** A. Adegboyega, A.T. Wiggins, O. Obielodan, M. Dignan, N. Schoenberg

**Affiliations:** 1 University of Kentucky College of Nursing, 751 Rose Street, Lexington, KY 40536-0232 USA; 2 University of Kentucky College of Public Health, Lexington, USA; 3 Prevention Research Center, University of Kentucky College of Medicine, Lexington, KY USA; 4 Center for Health Equity Transformation, College of Medicine, University of Kentucky, 468 Healthy Kentucky Research Building, Lexington, KY 40536 USA; 5 Department of Behavioral Science, College of Medicine, University of Kentucky, Lexington, USA

**Keywords:** Religiosity, Fatalism, Temporal orientation, Acculturation, Beliefs, Cancer screening

## Abstract

**Background:**

Beliefs influence cancer screening. However, there are conflicting findings about how belief influence cancer screening among Black adults. The aim of this study was to evaluate the relationships between beliefs (religiosity, fatalism, temporal orientation, and acculturation) and cervical, breast, and colorectal cancer screening behaviors among African Americans and sub-Saharan African immigrants.

**Methods:**

We conducted a cross-sectional survey of 73 African American and 59 English speaking Sub-Saharan immigrant adults recruited from Lexington and surrounding cities in Kentucky. Data collected included sociodemographic variables, cancer screening behaviors, and several instruments that characterize beliefs, including religiosity, fatalism, temporal orientation, and acculturation.

**Results:**

Participants’ mean age was 43.73 years (SD = 14.0), 83% were females, and 45% self-identified as sub-Saharan immigrants. Based on eligibility for each screening modality, 64% reported having ever had a Pap test, 82% reported ever having mammogram, and 71% reported ever having a colonoscopy. Higher education (OR = 2.62, 95% CI = 1.43—4.80) and being insured (OR = 4.09, 95% CI = 1.10 – 15.18) were associated with increased odds of cervical cancer screening (pap test), while cancer fatalism (OR = 0.24, 95% CI = 0.07 – 0.88) was associated with decreased odds. Increased age (OR = 1.57, 95% CI = 1.06 – 2.32) and reduced present orientation (OR = 0.42, 95% CI = 0.22 – 0.80) were associated with receipt of a mammogram. Nativity was the only factor associated with colonoscopy screening. Compared to African Americans, sub-Saharan African immigrants were 90% less likely to have had a colonoscopy (OR = 0.10, 95% CI = 0.02 – 0.66).

**Conclusion:**

This study contributes to the existing literature by confirming that beliefs are important in cancer screening behaviors among African American and sub-Saharan African immigrants. These findings should inform the development of cancer control and prevention programs for Black adults.

**Trial registration:**

US National Library of Science identifier NCT04927494. Registered June 16, 2021, www.clinicaltrials.gov

## Background

In 2022, there will be approximately 224,080 new cancer cases and 73,680 cancer deaths among Black people in the United States (U.S.) [1]. Collectively, Black individuals have the highest death rates and shortest survival for most cancers, when compared with other racial/ethnic groups in the U.S. [2]. These disparities also exist among sub-Saharan African immigrants [3, 4]; a sub-group often grouped with native born and other foreign Black populations. The sub-Saharan African immigrant population is one of the fastest growing immigrant populations in the U.S. [5]; and makes up 38% of the Black immigrant population which has increased over five times in four decades [2]. Despite the growing presence of this population, little is known about the cancer burden among sub-Saharan Africans [6]. Available sparse data tends to be aggregated with the overall Black population and may mask important differences that can inform cancer prevention and control efforts among specific sub-groups [7]. Sub-Saharan African immigrants to the U.S. may encounter similar facilitators and challenges to obtaining cancer screening as native-born Blacks, however, it is likely that these two groups maintain differing beliefs that may account for variations in behavior.

### Cancer disparities among Black individuals

Racial disparities in cancer have been well documented [1, 2], and have been found to be multifactorial and include advanced stage at diagnosis, lack of screening and follow up, and lack of access to cancer care and treatment [8–10]. It has been estimated that nearly 20% of the disparity in colorectal cancer death rates is attributable to lower screening rates among African Americans compared to Whites [11]. Although, cervical cancer death rates have reduced steadily because of early detection due to screening, data reveal disparities in screening for nearly all cancers [2, 12]. In addition, immigrant populations in the U.S. have lower cancer screening rates when compared to nonimmigrant population [3, 4, 13]. Researchers have suggested differences in beliefs as one of the reasons for the disparities in cancer incidence, mortality, and survival rates [14, 15]. Research show a correlation between beliefs and health behaviors, including the receipt of cancer screening [2, 16, 17].

### Beliefs associated with cancer screening

For this study, based on existing research, we conceptualized beliefs associated with cancer screening behaviors as comprised of religiosity, fatalism, temporal orientation, and acculturation [18–20]. Religiosity is defined as the presence of institutional organization and affiliation, expressions of particular beliefs, and rituals rooted in beliefs of the supernatural or divine [21]. Religiosity may help to develop an appreciation for one's body because of viewing the body as having spiritual as well as material significance and, therefore, support undertaking preventive health screenings [22, 23]. Some studies report positive association between religiosity [24] and cancer prevention while others did not find an association [25]. Fatalism is the tendency to believe that events are inevitable, and nothing can be done to change these events [26]. Fatalism has been found to negatively influence health promoting practices such as cancer screening among African Americans [27]. Fatalism is also a major factor that affects cancer preventive screening among sub-Saharan African immigrants, specifically breast and cervical cancers [7]. Health temporal orientation is the time perspective with which one makes health decisions and how an individual values distant outcomes relatively to present ones [28, 29]. An individual’s time perspective is influenced by his/her culture to some extent. Studies have reported that preventive health behaviors such as health screenings appear to be related to future time orientation [30]. Individuals with present orientation tend to focus on immediate behavior and its outcomes as against future consequences of behavior. These individuals may be less likely to engage in behaviors that will not yield immediate benefits [31]. Researchers have reported that Blacks are more likely to endorse present-time orientation when compared to Whites [32, 33]. The present-time orientation has been shown to negatively influence Black individuals attitude towards cancer screening [34]. Acculturation refers to the process in which an individual adopts or adheres to attitudes, beliefs, practices, or behaviors congruent with that of the dominant culture [35]. Acculturation has the potential to lead to adoption of positive or negative behaviors from the dominant culture [36]. Researchers have reported associations between lower levels of acculturation and lower use of cancer screening among immigrant groups in the U.S. [19, 36].

Less is known about beliefs and cancer, specifically, concerning beliefs relevant to African Americans and sub-Saharan African immigrants that explain preventive behaviors such as cancer screening. Knowing the influence of beliefs on cancer screening behaviors will further improve our understanding of cancer prevention and control behaviors and ultimately inform cancer prevention and enhance the design of successful health promotion interventions. Therefore, we examined religiosity, fatalism, temporal orientation, and acculturation and their association with cervical, breast, and colorectal cancer screening behaviors among African Americans and sub-Saharan African immigrants.

#### Theoretical framework

The analysis was informed by the Social Cognitive Theory (SCT) [37]. The SCT suggests that human motivation and action are extensively regulated by forethought. The SCT posits that behavior is the product of the dynamic interplay of personal, behavioral, and environmental influences [38]. The associations between religiosity, fatalism, temporal orientation, and acculturation and cancer-screening concur with the SCT model that provides an organizing framework in which beliefs and cultural embeddedness may shape the ways in which efficacy beliefs are developed to complete health behaviors.

## Methods

### Sampling method and sample size

We used a non-probability sampling method and a cross-sectional design. The study sample was a convenience sample of African American and sub-Saharan immigrant men and women living in Lexington and its surrounding cities in KY, which was drawn from a larger study aimed to examine general awareness and cultural factors related to cancer control and prevention among this population. Participants (*N* = 132) who were eligible for cervical, breast or colorectal cancer screening or whom complete data on variables of interest were available were included in the analyses.

### Study setting and recruitment

Research staff recruited eligible participants from the community using word of mouth and flyers placed at churches and social organizations. Through an online search, we identified social organizations and churches with Black congregants within our target cities. We contacted leaders of the organizations and churches and sort permission to inform their members of the on-going study and share flyers. Individuals who were interested in participating were screened for eligibility; eligible participants received a link via email to complete the electronic survey.

In addition, participants were recruited online using the University’s participant recruitment services between November 2020 and October 2021.The study was advertised via the University’s Center for Clinical Translational Science research recruitment website, participants who were interested in participating and learning more about the study completed a webform and were contacted by the research team to determine eligibility. Eligible participants were 18 years or older, self-identified as African American or sub-Saharan African immigrant, live in a mid-town southeastern city and its surrounding cities with no plans to relocate out of the area in the next 18 months and showed a willingness and ability to participate. The University Office of Research Integrity provided expedited review and approved the study with a waiver of informed consent documentation. Participants received a link to a self-administered online survey; we included a cover letter at the beginning of the survey to explain the purpose of the study and to let participants know that the survey was voluntary. Participants indicated their consent by proceeding to complete the survey. Individuals recruited in person had the option to complete the survey on paper. Participants received a $20 e-gift card as compensation for their time.

### Survey measures

The participant survey required approximately 20 min to complete and included questions on sociodemographics, cancer screening behaviors, religiosity, fatalism, temporal orientation, and acculturation. Sociodemographic variables include self-reported age, sex, ethnicity, education level, insurance status, and health literacy.

### Outcome variables

#### Receipt of cervical cancer screening (female > 21 years)

To assess ever having received cervical cancer screening, participants were asked if they had ever had a Pap test, with response options of “yes”, “no”, or “don’t know”. Women who answered, “don’t know” were classified with women who answered no to dichotomize the response.

#### Receipt of breast cancer screening (female > 40 years)

To assess ever having had a breast cancer screening, participants were asked if they had ever had a mammogram, with response options of “yes”, “no”, or “don’t know”. Women who answered, “don’t know” were classified with women who answered no to dichotomize the response.

#### Receipt of colorectal cancer screening (participants > 50 years)

To assess ever having had a colorectal cancer screening, participants were asked if they had ever done a colonoscopy, with response options of “yes” or “no”.

### Independent variables

Cancer fatalism, health temporal orientation, religiosity, and acculturation were independent variables and measured using the following validated instruments.

#### Cancer fatalism

Two questions from the validated items from the Health Information National Trends Survey (HINTS) were used to assess cancer fatalism [39]. We asked participants to indicate the extent to which they agreed with the two sentences, “There’s not much you can do to lower your chances of getting cancer,” and “When I think of cancer, I automatically think of death.” Response options for these questions were: “strongly disagree,” “disagree,” “not sure,” “agree,” and “strongly agree.” For our analysis, “strongly disagree/disagree” and “strongly agree/agree” responses were combined for each item. Responses were recoded so that higher values represented greater agreement with the belief.

#### Health temporal orientation

Perception of time as being in the present or future was assessed with five items on a 5-point Likert scale (strongly disagree- 1) to (strongly agree -5) to capture participants’ responses. Present orientation was based on four items, while one item measured future orientation [32, 40]. Sample items include, “It is important for me to focus on health issues that I am facing right now, not those that I might develop in the future” and “I often think about how my actions today will affect my health when I am older.”

#### Religiosity

Religiosity was measured with the religious commitment inventory (RCI) [41]. The RCI [41] has 10 items that measure the degree to which a person adheres to their religious values, beliefs, and practices and uses them in daily living. Participants rate their agreement with each item on a 5-point rating scale from 1 (not at all true of me) to 5 (totally true of me). Sample items include, “I enjoy spending time with others of my religious affiliation” and “Religion is especially important to me because it answers many questions about the meaning of life.” Higher scores indicate higher religious commitment.

#### Acculturation

Acculturation was assessed with the African American Acculturation Scale [42]. The scale has 10 items, respondents answered questions on a 4-pointLikert scale format, with responses ranging from 1 (i.e., Strongly disagree) to 4 (i.e., Strongly agree). Sample items include, “Socially, you feel less at ease with Whites than Blacks” and the highest proportions of your friends that you usually see these days are Black.” Scale scores represented the mean of items comprising the measure with lower scores representing higher levels of acculturation towards the dominant culture. High scores represent a more traditional African American cultural background (lower degree of acculturation); conversely, low scores suggest greater acculturation to majority American society (higher degree of acculturation).

### Data analysis

Descriptive analysis, including means and standard deviations or frequency distributions summarized variables. Unadjusted associations between demographic, belief factors, and each screening modality were accomplished using the two-sample t-test, chi-square test of association or Mann–Whitney U test. Multiple logistic regression models were used to evaluate adjusted associations. For each model, potential predictor variables included age, sex (included in the screening for colorectal cancer model only), nativity, education, insurance status and only the cultural factors that were significant in the bivariate analysis, due to sample size. The Hosmer–Lemeshow test was used to evaluate model fit for each regression model. All analysis was conducted using SAS (Cary, NC), version 9.4, with an alpha of 0.05 throughout.

## Results

One hundred and ninety-eight participants completed the survey. We included responses from 132 participants who were eligible for cervical, breast or colorectal cancer screening. We excluded individuals younger than 21 years of age because current guidelines do not recommend cancer screening for the screenings under investigation. Table 1 describes participant characteristics. Consistent with the age and sex eligibility for these screening methods, the average age was 43.7 years (SD = 13.6), and the majority were female (83%). Slightly more than half of the participants were African Americans (55%), while all others were sub-Saharan African immigrants. The top countries represented in the immigrants’ sample were Nigeria, Cameroon, and Congo. Overall, the sample had adequate health literacy, with less than one-quarter reporting more than occasional help needed with reading health-related instructions, pamphlets, and other written materials. More than half of the participants reported a college or graduate degree (51%), and over three-quarters were insured (77%). Average religiosity scores were 34.5 (SD = 11.0, potential range 10–50), indicating moderate religious commitment. (A score of 38 or higher indicates high religious commitment [41]). For cancer fatalism items, one-in-six (16%) agreed there’s not much you can do to lower your chances of getting cancer, and more than one-quarter (27%) agreed when they think of cancer, they automatically think of death. On average, participants had high scores for future orientation (*M* = 4.0, SD = 0.9, potential range 1–5), and moderate scores for present orientation (*M* = 9.4, SD = 3.5, potential range 4–18). Based on a potential range or 1–4, with lower scores reflecting higher acculturation, mean acculturation scores were also moderate (*M* = 2.3, SD = 0.5). Among those eligible for each screening modality, almost two third had ever had a Pap test (64%), more than three quarter had ever had a mammogram (82%), and nearly three-quarters had ever had a colonoscopy (71%).

**Table 1:** Descriptive summary of demographic characteristics and beliefs among participants (*N* = 132)

Variable	Potential range	*Mean* (SD); range or *n* (%)	Nativity		*p*
*Demographics*			Sub-Saharan African immigrant (*n* = 59)	African American (*n* = 73)	
Age	18–75	43.3 (14.0);18–75	43.7 (13.0); 19–70	43.0 (14.8); 18–75	.75
Sex					.029
Male		23 (17.4%)	15 (25.4%)	8 (11.0%)	
Female		109 (82.6%)	44 (74.6%)	65 (89.0%)	
Education					
High school		31 (23.5%)	7 (11.9%)	24 (32.9%)	
Some college		34 (25.8%)	13 (22.0%)	2 (28.8%)	
College graduate		32 (24.2%)	16 (27.1%)	16 (21.9%)	
Graduate school		35 (26.5%)	23 (34.0%)	12 (16.4%)	
Insurance status					.24
Insured		101 (76.5%)	48 (81.4%)	53 (72.6%)	
Uninsured		31 (23.5%)	11 (18.6%)	20 (27.4%)	
*Beliefs*					
Religiosity	10–50	34.5 (11.0)	38.4 (8.2)	31.5 (12.0)	< .001
*Fatalism items*					
There’s not much you can do to lower your chances of getting cancer					.21
Agree		21 (15.9%)	12 (20.3%)	9 (12.3%)	
Disagree		111 (84.1%)	47 (79.7%)	64 (84.7%)	
When I think of cancer, I automatically think of death					.97
Agree		36 (27.3%)	16 (27.1%)	20 (27.4%)	
Disagree		96 (72.7%)	43 (72.9)	53 (72.6%)	
Temporal orientation					
Future orientation	1–5	4.0 (0.9)	4.0 (1.1)	4.0 (0.8)	.73
Present orientation	4–20	9.4 (3.5)	8.9 (3.6)	9.9 (3.5)	.11
Acculturation	1–4	2.3 (0.5)	2.2 (0.5)	2.4 (0.5)	.004
*Screening*					
Pap screening (*n* = 109 eligible)					.97
Yes		70 (64.2%)	29 (64.4%)	41 (64.1%)	
No		39 (35.8%)	16 (35.6%)	23 (35.9%)	
Mammography screening (*n* = 49 eligible)					.92
Yes		40 (81.6%)	17 (80.9%)	23 (82.1%)	
No		9 (18.4%)	4 (19.0%)	5 (17.9%)	
Colonoscopy screening (*n* = 45 eligible)					.001
Yes		32 (71.1%)	10 (47.6%)	22 (91.7%)	
No		13 (28.9%)	11 (52.4%)	2 (8.3%)	

In testing for associations among the sociodemographic characteristics and belief items and nativity, compared to Sub-Saharan African immigrants, a higher proportion of African American participants were female (89.0% vs. 74.6%, *p* = 0.029; see Table 1). Immigrant participants had higher educational attainment (*p* < 0.001) and had higher scores on the religious commitment scales (*M* = 38.4, SD = 8.2 vs. *M* = 31.5, SD = 12.0, *p* < 0.001), and slightly lower acculturation scores (*M* = 2.2, SD = 0.5 vs. *M* 2.4, SD = 0.5, *p* = 0.004), compared to African American participants. A higher proportion of African American participants had ever had a colonoscopy (91.7% vs. 47.6%, *p* = 0.001). The two groups did not differ on age, insurance status, either of the fatalism items, temporal orientation, Pap, or mammography screening.

### Determinants of cervical cancer screening

Among women eligible for Pap test (*n* = 109), those who reported ever having a Pap test were significantly older (*M* = 42.8, SD = 11.7 vs. *M* = 34.3, SD = 11.6; *p* < 0.001), had higher levels of education (*p* < 0.001) and were more likely to report being insured (89% vs. 51%, *p* < 0.001; see Table 2) compared to those who had not. The beliefs associated with receipt of a Pap test included cancer fatalism (*p* < 0.001), present orientation (*p* < 0.001), and acculturation (*p* < 0.001). Compared to women who had not had a Pap test, those who had been screened had fewer fatalistic views about cancer and death (10% vs. 49%), lower scores for present orientation (*M* = 8.8, SD = 3.3 vs. *M* = 11.0, SD = 3.3), and higher acculturation scores (*M* = 2.2, SD = 0.5 vs. *M* = 2.5, SD = 0.5).

**Table 2:** Unadjusted and adjusted associations among demographic, beliefs and ever having had Pap test among eligible study participants (*n* = 109)

Ever had a Pap test						
	Unadjusted		p	Adjusted (*n* = 108)		*p*
	Yes (*n* = 70) *Mean* (SD); or *n* (%)	No (*n* = 39) *Mean* (SD); or *n* (%)		*Odds ratio* (OR)	95% CI for OR	
Age	42.8 (11.7)	34.3 (11.6)	< .001	1.03	0.98 – 1.09	.19
Nativity						
Sub-Saharan African immigrant	29 (41.4%)	16 (41.0%)	.97	0.29	0.08 – 1.04	.056
African American	41 (58.6%)	23 (59.0%)		1.00		
Education			.001	2.62	1.43 – 4.80	.002
High school	11 (15.7%)	17 (43.6%)				
Some college	17 (24.3%)	15 (38.5%)				
College graduate	3 (34.3%)	5 (12.8%)				
Graduate school	18 (25.7%)	2 (5.1%)				
Insurance status			< .001	4.09	1.10 – 15.18	.036
Insured	62 (88.6%)	20 (51.3%)				
Uninsured	8 (11.4%)	19 (48.7%)				
Religiosity (10–50)	34.7 (11.1)	30.5 (9.8)	.051	1.02	0.96 – 1.09	.48
Cancer fatalism items						
There’s not much you can do to lower your chances of getting cancer						
Agree	11 (15.7%)	3 (7.7%)	.23			–
Disagree	59 (84.3%)	36 (92.3%)				
When I think of cancer, I automatically think of death						
Agree	7 (10.0%)	19 (48.7%)	< .001	0.24	0.07 – 0.88	.032
Disagree	63 (90.0%)	20 (51.3%)				
Temporal orientation						
Future orientation (1–5)	4.0 (1.0)	3.8 (0.7)	.41			–
Present orientation (4–20)	8.8 (3.3)	11.0 (3.3)	< .001	0.96	0.80 – 1.15	.63
Acculturation (1–4)	2.2 (0.5)	2.5 (0.5)	.020	0.73	0.24 – 2.25	.58

The regression model was restricted to demographic characteristics and those beliefs significant in the bivariate (unadjusted) analysis

The logistic regression model including key demographics and cultural factors that were significant in the bivariate analysis was significant overall (X^2^ = 49.2, *p* < 0.001) and the Hosmer–Lemeshow test was non-significant (*X*^*2*^ = 7.0, *p* = 0.53), indicating no issues with model fit. Education (*p* = 0.002), insurance status (*p* = 0.036) and cancer fatalism (*p* = 0.032) remained significant in the adjusted analysis. Every increase in the level of education was associated with an over two-fold increase in the odds of having ever had a Pap test (*OR* = 2.62, 95% CI = 1.43 – 4.80). Insured women were over 4 times more likely to have ever had a Pap, compared to the uninsured (*OR* = 4.09, 95% CI = 1.10 – 15.18). Women who agreed that having thoughts about cancer automatically made them think of death, had 0.24 times the odds to have had a Pap test (*OR* = 0.24, 95% CI = 0.07 – 0.88). Age, nativity, religiosity, present orientation, and acculturation were not significant in the adjusted analysis.

### Determinants of mammography

Among women who were eligible for mammography (*n* = 49), those who reported ever having a mammogram were significantly older (*M* = 52.9, SD = 8.2 vs. *M* = 44.4, SD = 3.2, *p* < 0.001; see Table 3) and had lower present orientation scores (*M* = 8.4, SD = 2.9 vs. *M* = 12.3, SD = 3.2, *p* < 0.001) compared to women who had never had a mammography. Religiosity, cancer fatalism, future orientation, and acculturation, were not associated with mammography. The overall logistic regression model for mammography screening was significant (*X*^*2*^ = 29.2, *p* < 0.001), and these same two variables remained significant. Every one-year increase in age was associated with a 1.6 times increase in the odds of mammogram (OR = 1.57, 95% CI = 1.06 – 2.32, *p* = 0.024), while a one-point increase in present orientation was associated with a 58% decrease in the likelihood of screening (*OR* = 0.42, 95% CI = 0.22 – 0.80, *p* = 0.009). Nativity, education, and insurance status were not significant in the adjusted model. The Hosmer–Lemeshow test for this model was non-significant, indicating no issues with model fit (*X*^*2*^ = 2.2*, p* = 0.95).

**Table 3:** Unadjusted and adjusted associations among demographic, beliefs and having ever had a mammogram among eligible study participants (*n* = 49)

	Ever had a mammogram					
	Unadjusted		p	Adjusted (*n* = 49)		*P*
	Yes (*n* = 40) *Mean* (SD); or *n* (%)	No (*n* = 9) *Mean* (SD); or *n* (%)		*Odds ratio* (OR)	95% CI for OR	
Age	52.9 (8.2)	44.4 (3.2)	< .001	1.57	1.06 – 2.32	.024
Nativity			.92			.66
Sub-Saharan African immigrant	23 (57.5%)	5 (55.6%)		1.75	0.14 – 21.92	
African American	17 (42.5%)	4 (44.4%)		1.00		
Education			.53	1.21	0.40 – 3.70	.73
High school	9 (22.5%)	2 (22.2%)				
Some college	8 (20.0%)	2 (22.2%)				
College graduate	10 (25.0%)	4 (44.4%)				
Graduate school	13 (32.5%)	1 (11.1%)				
Insurance status			.64	0.05	< 0.01 – 219.38	.49
Insured	33 (82.5%)	8 (88.9%)				
Uninsured	7 (17.5%)	1 (11.1%)				
Religiosity (10–50)	37.6 (11.0)	37.9 (8.4)	.95			–
Cancer fatalism items						
There’s not much you can do to lower your chances of getting cancer						
Agree	6 (15.0%)	1 (11.1%)	.99			–
Disagree	34 (85.0%)	8 (88.9%)				
When I think of cancer, I automatically think of death			.57			–
Agree	3 (7.5%)	1 (11.1%)				
Disagree	37 (92.5%)	8 (88.9%)				
Temporal orientation						
Future orientation (1–5)	3.9 (1.1)	3.6 (1.2)	.41			–
Present orientation (4–20)	8.4 (2.9)	12.3 (3.2)	< .001	0.42	0.22 – 0.80	.009
Acculturation (1–4)	2.2 (0.4)	2.3 (0.5)	.57			–

The regression model was restricted to demographic characteristics and those beliefs significant in the bivariate (unadjusted) analysis

### Determinants of colonoscopy for colorectal cancer screening

There were 45 men and women eligible for colonoscopy screening for colorectal cancer screening. Compared to those who had not had a colonoscopy, a higher proportion of those who had were African American (68.7% vs. 15.4%), and were insured (87.5% vs. 61.5%, *p* = 0.049; see Table 4). Religiosity, fatalism, temporal orientation, and acculturation were not associated with colonoscopy uptake. In the adjusted analysis, the overall logistic regression model was significant (*X*^*2*^ = 15.7, *p* = 0.008). The only significant predictor was nativity (*p* = 0.017); compared to African Americans, sub-Saharan African immigrants were 90% less likely to have had a colonoscopy (*OR* = 0.10, 95% CI = 0.02 – 0.66). Age, sex, education, and insurance status were not significant in the adjusted model. Again, the Hosmer–Lemeshow test was non-significant (*X*^*2*^ = 6.4, *p* = 0.50), so there was no evidence of concern with model fit.

**Table 4:** Unadjusted and adjusted associations among demographic, beliefs and having ever had a colonoscopy among eligible study participants (*n* = 45)

	Ever had a colonoscopy					
	Unadjusted		p	Adjusted (*n* = 62)		*p*
	Yes (*n* = 32) *Mean* (SD); or *n* (%)	No (*n* = 13) *Mean* (SD); or *n* (%)		*Odds ratio* (OR)	95% CI for OR	
Age	60.3 (6.8)	56.1 (5.1)	.049	1.13	0.95 – 1.35	.17
Sex			.28			.47
Male	14 (43.8%)	8 (61.5%)		0.51	0.08 – 3.16	
Female	18 (56.2%)	5 (38.5%)		1.00		
Nativity			.002			.017
Sub-Saharan African immigrant	10 (31.3%)	1 (84.6%)		0.10	0.02 – 0.66	
African American	11 (68.7%)	2 (15.4%)		1.00		
Education			.57	1.57	0.68 – 3.64	.30
High school	5 (15.6%)	2 (15.4%)				
Some college	6 (16.8%)	2 (15.4%)				
College graduate	8 (25.0%)	2 (15.4%)				
Graduate school	13 (40.6%)	7 (53.8%)				
Insurance status			.049			.38
Insured	28 (87.5%)	8 (61.5%)		2.39	0.35 – 16.49	
Uninsured	4 (12.5%)	5 (38.5%)		1.00		
Religiosity (10–50)	41.2 (10.0)	39.3 (8.3)	.54			–
Cancer fatalism items						
There’s not much you can do to lower your chances of getting cancer			.25			–
Agree	7 (21.9%)	5 (38.5%)				
Disagree	25 (78.1%)	8 (61.5%)				
When I think of cancer, I automatically think of death			.10			–
Agree	7 (21.9%)	6 (46.1%)				
Disagree	25 (78.1%)	7 (53.9%)				
Temporal orientation						
Past orientation (1–5)	4.3 (0.8)	3.8 (1.3)	.26			–
Present orientation (4–20)	8.8 (3.4)	9.3 (4.3)	.67			–
Acculturation (1–4)	2.3 (0.4)	2.3 (0.4)	.94			–

The regression model was restricted to demographic characteristics and beliefs significant in the bivariate (unadjusted) analysis

## Discussion

The purpose of this study was to examine beliefs (religiosity, fatalism, temporal orientation, and acculturation) and their association with cervical, breast, and colorectal cancer screening behaviors among African Americans and sub-Saharan African immigrants. Understanding beliefs relevant for cancer screening among Blacks may be important for designing culturally tailored behavioral interventions to promote screening and reduce cancer disparities experienced by these groups. This study highlighted several key findings.

Among participants eligible for each screening modality, 64% had ever had a pap test, 82% had ever had a mammogram, and 71% had ever had a colonoscopy. While mammogram completion rate in this study was higher than the Healthy People 2030 target of 77.1%, the cervical and colorectal cancer screening rates were below the Healthy People 2030 target of 84.3% and 74.4% respectively [43]. The disparity between the Healthy People 2030 target goal and the rates of screening completion among the study participants suggests that African American and sub-Saharan African immigrants in this study are at risk for late-stage detection due to underutilization of cancer screening and missed opportunities for early detection.

The sociodemographic correlates for having had a Pap test were higher level of education and being insured; correlates for having had a mammogram was older age; and correlates for colorectal cancer screening was nativity. The positive association between higher level of education and the adherence to Pap and colorectal cancer screening is in consonance with previous studies [44–46]. One possible explanation may be that those individuals with higher educational attainment have greater awareness about their cancer risk, which prompts adoption of healthy lifestyles, and more means to access screening. Furthermore, literature suggests that individuals with higher levels of education report greater interest, more knowledge about health issues, and are more likely to have health insurance and to belong to social networks that encourage preventive and healthy behaviors [47]. Educational attainment has been shown to affect health literacy which, in term, may influence full comprehension of health education materials [48].

In addition, we found that being insured was associated with having ever had a Pap test. This finding agrees with previous research that suggests that health insurance promotes clinical preventive service use [44, 46]. Health insurance increases access to medical care, which may provide opportunity for reminders or cues for preventive screening and adoption of healthy behaviors as well as reduction of risky behaviors based on a health provider’s recommendation [49].

We found that mammogram uptake increased with age. One possible reason for this may be that younger women have not yet initiated mammogram due to lack of provider’s recommendation while older women, who have received multiple recommendations have initiated the screening. Another possible reason may be related to shifting or ambiguous recommendations about the best age to start screening. In the US, major organization’s clinical guidelines differ in their recommendations about mammograms for women aged 40–50. For example, the U.S. Preventive Services Task Force recommends biennial screening for women 50–74 years with average risk [50] while the American Cancer Society recommends annual screening at 45–55 years of age and biennial screening at 55 years or older) [51].

We found that Sub-Saharan African immigrants were less likely to have had a colonoscopy. The screening rates found in this study suggest that Sub-Saharan African immigrants are disproportionately underusing colonoscopy screening and may be at risk for late-stage detection. In accordance with literature, Sub-Saharan African immigrants and other immigrants have lowest screening rates compared to native-born individuals [3, 4, 7, 52]. One possible reason for this may be that sub-Saharan African immigrants do not receive recommendation to screen for colorectal cancer or are unaware of the colorectal cancer screening guidelines. A literature review by Hurtado-de-Mendoza et al. found that African immigrants had lower cancer screening rates and lower awareness of screening recommendations compared with other populations [7]. A recent study among African immigrants in the U.S. using data from the National Health Interview Survey from 349 African-born immigrants found significant association between colorectal cancer screening and health insurance status, length of stay in the U.S., perceived health status, and having a usual place for medical care [53]. Although we found significant association between colorectal cancer screening and insurance status in bivariate analysis, but this was not significant in the adjusted model. With increased migration of Sub-Saharan African immigrants to the U.S, the factors underlying colorectal cancer screening disparities between Sub-Saharan African immigrants and other groups need to be better understood, in order to inform targeted interventions to close the gaps in screening rates and promote equitable access and benefits from screening.

Our results indicated that those endorsing fatalistic beliefs are generally less likely to obtain Pap screening; but there was no association with breast and colorectal cancer screening. Previous studies have shown that cancer fatalistic beliefs can influence cancer screening and prevention behaviors [26, 27, 54]. Fatalistic beliefs about surviving cancer may inhibit individuals from engaging in screening tests out of fear that a positive test result represents a death sentence [54]. It is unclear why there was an association between fatalistic beliefs and cervical screening but not the use of the other cancer screening modalities. Future research is needed to better understand and examine the potentially complex relationships among cancer screening and fatalistic beliefs.

The results of this study support the relationship between present orientation and decreased likelihood of completing mammogram. This finding is consistent with reports from previous research [28, 30, 33]. Individuals that are future oriented may be more likely to participate in preventive cancer screening because these individuals may see a greater value on the benefits of screening. To promote preventive screening, health researchers should consider health temporal orientation and other beliefs in the design of interventions for Black populations.

### Implications and recommendations

The findings from this study have several implications for public health and research. First, there is need for tailored culturally appropriate interventions targeted at promoting Pap and colorectal cancer screening among these populations. Culturally appropriate educational interventions are needed to promote cancer screening and address misconceptions about cancer prevention and survivorship. Future interventions to promote cancer screening should address unique fatalistic and temporal orientation beliefs peculiar to African American and sub-Saharan African immigrants through targeted educational interventions. Furthermore, there is need to increase cancer prevention and awareness efforts among younger individuals; specifically, younger Black women should be targeted for mammogram to promote early screening, which could lead to early detection and better treatment outcomes. Chapman and colleagues suggested that initiation of mammogram earlier than is presently recommended for the overall U.S. population by the USPSTF or the American Cancer Society can reduce mortality disparities and maintain acceptable benefit–harm tradeoffs for Black women [55].

Second, with an increase in migration of sub-Saharan immigrants to the U.S., this group should be targeted for colorectal cancer preventive education and screening promotion to close gaps in screening rates. Providing an expanded variety of screening options may lead to increased colorectal screening, as individuals will often choose screening options with fewer barriers and consistent with their preferences [56]. The findings from this study suggest that more effort is needed to provide screening recommendations and guidelines for colorectal cancer among Black individuals and particularly among sub-Saharan African immigrants.Third, additional research on the role of race, ethnicity, and nativity to foster better understanding of preventive screening among Black individuals is warranted. Insights into the beliefs, cultural factors, and barriers to healthcare among subgroups of Black individuals can be used to improve health outcomes and reduce disparities suffered by Blacks. Research that separates data among groups of U.S. Blacks and recognizes diversity in the Black population may uncover differing beliefs and facilitate efforts to reduce disparities and promote the health of all U.S. citizens.

### Limitations

Several study limitations should be considered in the interpretation of the study results. First, the study design was a cross-sectional research design, which does not reflect causation. Second, a convenience and non-probability sample was utilized in this study; the sample is skewed toward highly educated/insured compared to these two groups overall, and results may not be generalizable to African American and sub-Saharan African immigrant populations. Third, another limitation of this analysis was the modest sample size, which was further restricted for each model based on those who were appropriate for the screening modality. Future studies in this area will benefit from larger-scale, powered studies with increased inclusion of participants and robust sampling strategies Four, participants self-reported prior completion of screening, which may be subject to recall or social desirability bias. The dichotomization of Pap screening responses may bias the results. In addition, the survey scope was limited, there are other methods for cervical (human papillomavirus test) and colorectal cancer screening that were not assessed the question on cervical and colorectal screening may have excluded individuals who completed their screening using other methods.

## Conclusion

Comprehensive cancer burden data would be useful to inform cancer prevention and control interventions for Black immigrants and native U.S population. The rates of cervical and colorectal cancer screening in this sample are suboptimal, which indicates the need for intervention to promote early cancer detection, reduce cancer disparities, and improve cancer outcomes. This study contributes to the existing literature by confirming that certain beliefs are important in cancer screening behaviors among African American and sub-Saharan African immigrants. These findings should inform the development of cancer control and prevention programs for Black adults.

## Data Availability

The data that support the findings of this study are available from the corresponding author, upon reasonable request.
